# A prospective study on total protein, plant protein and animal protein in relation to the risk of incident chronic kidney disease

**DOI:** 10.1186/s12882-020-02079-y

**Published:** 2020-11-17

**Authors:** Sevda Alvirdizadeh, Emad Yuzbashian, Parvin Mirmiran, Shahryar Eghtesadi, Fereidoun Azizi

**Affiliations:** 1 Department of Nutrition, Science and Research Branch, Islamic Azad University, Tehran, Iran; 2 Nutrition and Endocrine Research Center, Research Institute for Endocrine Sciences, Shahid Beheshti University of Medical Sciences, P.O. Box: 19395-4763, Tehran, Iran; 3 Endocrine Research Center, Research Institute for Endocrine Sciences, Shahid Beheshti University of Medical Sciences, Tehran, Iran

**Keywords:** Kidney function, Total protein, Plant protein, Animal protein

## Abstract

**Background:**

The link between dietary protein intake and the risk of kidney dysfunction is always a challenging issue. This study aimed to investigate the relationship between total protein, plant protein, and animal protein intake with the risk of incident chronic kidney disease (CKD).

**Methods:**

This study was performed on 1639 adults aged ≥27 years who participated in the Tehran Lipid and Glucose Study. Dietary data were evaluated using a valid and reliable semi-quantitative food frequency questionnaire (FFQ). Total protein content, plant protein, and animal protein of each participant were calculated. Glomerular filtration rate (GFR) less than 60 mL / min / 1.73 m^2^ has been considered as the definition of CKD. Odds Ratio (OR) was calculated using logistic regression to show the association between the risk of incident CKD and dietary exposures.

**Results:**

After adjusting for age, sex, body mass index, smoking, total energy intake, total fiber intake, dietary fat, physical activity, diabetes, and hypertension, there was no significant association of total protein and animal protein consumptions with the incidence of CKD. After adjustment for confounders, compared with the lowest tertile of plant protein consumption, OR of incident CKD in the highest tertile was 0.29 (95% confidence interval [95% CI] 0.15 to 0.55) with a significant trend (*P* for trend < 0.001).

**Conclusion:**

The results of this study confirmed an inverse association between plant protein intake and the risk of incident CKD, which demonstrates the protective role of plant-based protein in a diet on kidney function.

## Background

Chronic kidney disease (CKD) is a progressive systemic disease with increasing prevalence at the global level, affecting more than 10% of the world’s population and half of the adults older than 70 years of age [1–3]. The prevalence of CKD in an Iranian population over the age of 20 years was 11.4% [4]. Late diagnosis of CKD can lead to end-stage renal failure, increased premature mortality, low quality of life, and enormous costs for the health system. Therefore, appropriate strategies to prevent CKD are the most important solutions [5, 6]. Various studies have shown that nutritional factors play an essential role in preventing the development and progression of CKD [7].

According to recent studies, a very low protein diet as a part of nutritional therapy have beneficial effects in slowing the progression of CKD [8–10]. A meta-analysis of randomized controlled trials suggested a protective effect of soy protein consumption on serum creatinine and serum phosphorus concentrations in pre-dialysis CKD patients [11]. However, limited studies have examined the impact of habitual intake of protein and its major sources, including plant- and animal- protein on the onset of CKD. In our previous study, we reported that higher consumption of total red meat and processed meat increased CKD risk, and substitution of red meat with protein from plant sources decreased the risk of CKD [12]. Another study reported associations of different dietary protein sources with the risk of incident CKD and reported that red and processed meat were adversely associated with CKD risk, while nuts, low-fat dairy products, and legumes were protective against the development of CKD [13]. Since total protein intake contains both animal and plant protein sources in the diet, this study aimed to investigate the relationship between total protein, as well as plant protein and animal protein with the risk of CKD.

## Methods

### Study population

**Fig. 1:**
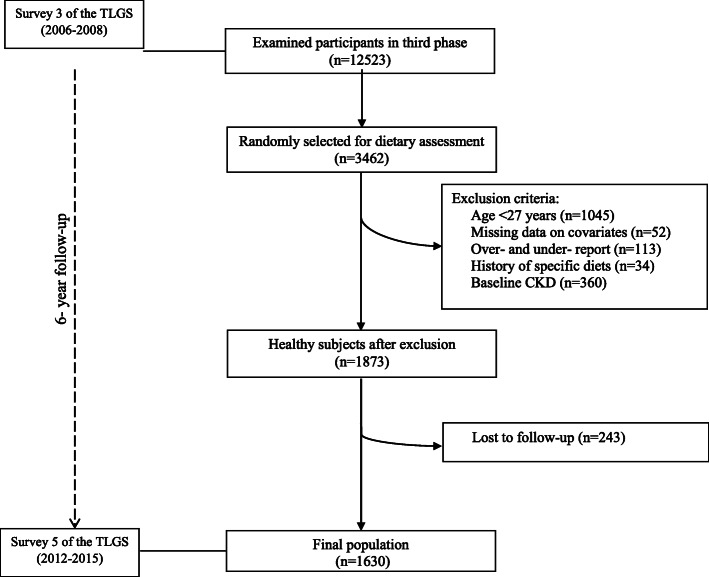
Flow chart of the Tehran Lipid and Glucose Study (TLGS) participants

This population-based study was conducted on the Tehran Lipid and Glucose Study (TLGS), which is an ongoing population-based prospective study [14]. The baseline survey of TLGS was initiated from 1999 to 2001, and the participants’ information was updated every 3-year. In the third survey, among the 12,523 participants with completed data, 3462 were randomly invited for dietary data collection. Of those, 2417 participants who were 27 years and older were included. We excluded participants if they had a history of myocardial infarction or stroke (*n* = 34) due to possible deviation from regular diet, reporting implausible daily energy intakes (< 800 or > 4200 kcal/d (*n* = 113), having missing data (*n* = 52). Some individuals fell into more than one exclusion category. To estimate the incidence of CKD, We excluded those who developed CKD at baseline (*n* = 360). After 6.1 years of follow-up through survey V (response rate: 87%), 1630 participants remained for final analysis (Fig. 1).

### Ethical consideration

The ethics committee of the Research Institute for Endocrine Sciences, Shahid Beheshti University of Medical Sciences approved the study protocol and informed written consent was obtained from all participants.

### Dietary assessment

The dietary intakes were assessed using a valid and reliable semi-quantitative FFQ by trained dietitians during face-to-face interviews [15–17]. Because the Iranian food composition table (FCT) is not completed yet, for the current study, we have to apply The United States Department of Agriculture FCT. We calculated total dietary protein, as well as animal protein and plant protein. The previous study approved the reasonable validity and reliability of used [17].

### Measurement of covariates

To estimate the physical activity of participants, modifiable activity questionnaire (MAQ) was used, and metabolic equivalent task (MET) minutes per week was calculated. In the previous study, the validity and reliability of the MAQ were reported [18]. We define low levels of physical activity as MET < 600 min/wk. [19].

Weight, height, and Blood pressure (BP) were measured by standard protocol [14]. Body mass index (BMI) was calculated as weight (kg) divided by square of height (m^2^). Blood samples of all participants were taken after overnight fasting. Fasting plasma glucose (FPG) and triglycerides (TGs) were assayed by enzymatic colorimetric method using glucose oxidase and glycerol phosphate oxidase, respectively. Inter- and intra-assay coefficients of variation (CVs) for FBS were less than 2%, and for TGs were 0.6 and 1.6%, respectively. The standard colorimetric Jaffe Kinetic reaction method was applied to measured serum creatinine with intra- and inter-assay CVs of below 3.1%. All analyses were performed using commercial kits (Pars Azmoon Inc., Tehran, Iran).

### Definition of terms

Participants with systolic blood pressure (SBP) ≥140, diastolic blood pressure (DBP) ≥90 mm-Hg or current therapy defined hypertension [20], and those with FPG ≥ 126 mg/dl or current treatment was defined as diabetes [21].

The following equation formula, which was proposed by Modification of Diet in Renal Disease (MDRD) was applied to calculate eGFR in ml/min/1.73m^2^ of body surface area [22]. Then, those with eGFR< 60 ml/min/1.73m^2^ were classified diagnosed CKD based on national kidney foundation guidelines [23].

$$ \mathrm{eGFR}=186\times \left(\mathrm{Serum}\ \mathrm{creatinine}\right)-1.154\times \left(\mathrm{Age}\right)-0.203\times \left(0.742\ \mathrm{if}\ \mathrm{female}\right)\times \left(1.210\ \mathrm{if}\ \mathrm{African}-\mathrm{American}\right). $$

### Statistical analysis

Data were analyzed using the Statistical Package for the Social Sciences program (SPSS) (version 16.0), and *P*-values< 0.05 were considered statistically significant. The characteristics values of participants were reported as mean (SD) for data with normal distribution and median (25th to 75th percentile) for data with non-normal distribution or percentages for qualitative variables.

Before conducting multivariable logistic models, as diabetes or hypertension could affect the contribution of plant protein, the interaction terms for [diabetes × plant protein] and [hypertension × plant protein] were used as separate inputs in our multivariable logistic regression models. There was no significant interaction between plant protein and diabetes (*P*-Value = 0.839) or plant protein and hypertension (*P*-Value = 0.345). Dietary total protein, animal protein, and plant protein were categorized into three groups according to the tertiles of the distribution among the total population. Odds ratio (OR) and 95% confidence intervals (CIs) for the incidence of CKD according to tertiles of dietary exposure was assessed with multivariable logistic regression models. In this analysis, the first tertile of dietary exposure was considered as the reference group. Five models were considered to adjust potential confounders; Model 1, crude (without any covariate); model 2, age (continuous), sex (male/female), smoking (yes/no), BMI (continuous), total energy intake (continuous), physical activity (low, moderate, heavy); model 3, percent of energy from total fat (continuous), total fiber (continuous) in addition to cofounders in model 2; model 4, diabetes mellitus (yes/no), hypertension (yes/no), in addition to cofounders in model 3. Furthermore, model 5 was adjusted for FBS (continuous) and systolic BP (continuous) instead of diabetes mellitus and hypertension in addition to confounders in model 3.

## Results

In this study, 1630 adults were followed up for 6.1 years. After follow-up, 220 patients with CKD were found.

The baseline characteristics and dietary intakes of the participants categorized in three groups according to tertiles of dietary total protein intake are shown in Table 1. Subjects with higher intakes of total protein were mostly men. They also had more energy intake and more animal and plant protein intake. However, subjects with more consumption of total protein had less energy percent from carbohydrates and total fats.

**Table 1:** Baseline characteristics and dietary intakes of participants according to tertiles of the total protein intake

characteristics	Tertiles of the total protein			
	T1(*n* = 544)	T2(*n* = 542)	T3(*n* = 544)	*P* for trend
Age (years)	43.3 ± 11.3	42.8 ± 10.9	42.4 ± 11.4	0.868
Male (%)	44.3%	47.2%	57.0%	< 0.001
Body mass index (kg/m ^2^)	27.4 ± 4.5	27.6 ± 4.7	27.8 ± 4.4	0.595
Current smoker (%)	9.6%	11.3%	10.5%	0.620
Low physical activity (%)	68.2%	65.2%	67.1%	0.664
Fasting blood sugar (mg/dl)	91.2 ± 21.1	92.4 ± 22.3	93.5 ± 23.1	0.215
Systolic blood pressure (mm Hg)	111.7 ± 15.5	112.1 ± 16.7	113.4 ± 16.2	0.379
Diastolic blood pressure (mm Hg)	74.0 ± 10.1	74.1 ± 11.2	74.6 ± 10.5	0.352
Triglyceride (mg/dL)	129.0 (91.0–183.7)	127.5 (86.0–184.2)	134.0 (94.0–201.0)	0.361
eGFR (ml/min/1.73 m ^2^)	73.2 ± 8.2	73.9 ± 9.1	73.9 ± 8.5	0.380
Cr (mg/dL)	1.02 ± 0.13	1.02 ± 0.14	1.05 ± 0.14	0.339
Diabetes (%)	8.6%	7.9%	7.9%	0.658
Hypertension (%)	13.2%	11.8%	12.5%	0.714
**Daily intakes**				
Total energy (Kcal)	1621 ± 364	2224 ± 443	3111 ± 745	< 0.001
Total protein (%energy)	12.75 ± 2.16	13.68 ± 2.14	14.75 ± 2.50	< 0.001
Total protein (gr)	50.36 ± 9.83	74.23 ± 6.43	114.44 ± 29.42	< 0.001
Animal protein (gr/1000 kcal)	16.2 ± 5.9	18.5 ± 6.0	20.9 ± 8.1	< 0.001
Animal protein (gr)	25.43 ± 8.33	39.43 ± 9.18	63.05 ± 26.65	< 0.001
Plant protein (gr/1000 kcal)	15.5 ± 3.6	15.8 ± 3.7	16.5 ± 4.9	< 0.001
Plant protein (gr)	24.92 ± 7.42	34.79 ± 8.99	51.38 ± 21.18	< 0.001
Total fiber (gr/1000 kcal)	16.1 ± 6.9	17.01 ± 6.7	17.5 ± 6.9	0.224
Carbohydrate (%energy)	58.1 ± 7.6	57.6 ± 6.8	57.5 ± 7.3	0.048
Total fat (%energy)	31. ± 7.7	31.4 ± 6.9	30.5 ± 6.5	0.023

In all five models, plant protein intake was associated with 70% decreased incidence of CKD (Table 2). After adjustment for age, sex, BMI, smoking, total energy intake, physical activity, total fat and fiber intake, diabetes, and hypertension the OR for participants in the lowest compared with those in the highest tertile of plant protein intake was 0.29 (95%CI: 0.15 to 0.55). In model five diabetes and hypertension were replaced with FBS and systolic BP and OR for participants in the highest compared with those in the lowest tertile of plant protein intake was 0.28 (95%CI: 0.14 to 0.53). A significant decreasing linear trend was noted across tertiles of plant protein intake for risk of incident CKD (*P* for trend< 0·001).

**Table 2:** Odds ratio (95% confidence intervals) of incident chronic kidney disease according to tertiles of dietary exposures

	Tertiles of dietary intake			
	T1	T2	T3	*P* for trend
Total Protein	*n* = 544(CKD = 82)	*n* = 542(CKD = 66)	*n* = 544(CKD = 71)	
Model 1	1.00	0.75 (0.52–1.09)	0.79 (0.54–1.14)	0.197
Model 2	1.00	0.67 (0.44–1.03)	0.62 (0.34–1.12)	0.090
Model 3	1.00	0.67 (0.44–1.02)	0.59 (0.32–1.07)	0.066
Model 4	1.00	0.67 (0.44–1.02)	0.59 (0.32–1.08)	0.071
Model 5	1.00	0.65 (0.43–1.00)	0.59 (0.32–1.08)	0.067
Plant Protein	*n* = 543(CKD = 90)	*n* = 544(CKD = 70)	*n* = 543(CKD = 59)	
Model 1	1.00	0.80 (0.56–1.14)	0.61 (0.42–0.90)	0.012
Model 2	1.00	0.63 (0.42–0.94)	0.41 (0.23–0.71)	0.001
Model 3	1.00	0.55 (0.36–0.84)	0.29 (0.15–0.55)	< 0.001
Model 4	1.00	0.55 (0.36–0.83)	0.29 (0.15–0.55)	< 0.001
Model 5	1.00	0.55 (0.36–0.83)	0.28 (0.14–0.53)	< 0.001
Animal protein	*n* = 544(CKD = 73)	*n* = 542(CKD = 74)	*n* = 544(CKD = 72)	
Model 1	1.00	0.95 (0.66–1.37)	0.91 (0.62–1.32)	0.626
Model 2	1.00	0.93 (0.63–1.38)	0.93 (0.59–1.46)	0.745
Model 3	1.00	0.94 (0.64–1.38)	0.91 (0.57–1.44)	0.686
Model 4	1.00	0.94 (0.63–1.38)	0.91 (0.57–1.45)	0.699
Model 5	1.00	0.94 (0.63–1.38)	0.91 (0.57–1.44)	0.681

*P* for trend across tertiles calculated with the exposure modelled as a continuous variable

Model 1: crude

Model 2: adjusted for age, sex, smoking, total energy intake, physical activity, BMI

Model 3: additionally adjusted for total fiber intake and energy percent from fat

Model 4: additionally adjusted for diabetes mellitus and hypertension

Model 5: additionally adjusted for FBS and systolic BP instead of diabetes and hypertension

After excluding diabetes mellitus and hypertension cases, 1334 subjects remained and 163 patients with CKD were found at the end of study. As it is shown in Table 3, incidence of CKD was significantly decreased in higher intakes of plant protein in all 3 models. After adjustment for age, sex, BMI, smoking, total energy intake, physical activity, total fat and fiber intake, the OR for participants in the lowest compared with those in the highest tertile of plant protein intake was 0.26 (95%CI: 0.12 to 0.56). A significant decreasing linear trend was noted across tertiles of plant protein intake for risk of incident CKD (*P* for trend< 0.001).

**Table 3:** Odds ratio (95% confidence intervals) of incident chronic kidney disease according to tertiles of dietary exposures. (Diabetes mellitus and hypertension cases were excluded)

	Tertiles of dietary intake			
	T1	T2	T3	*P* for trend
Total Protein	*n* = 435(CKD = 60)	*n* = 449(CKD = 54)	*n* = 450(CKD = 49)	
Model 1	1.00	0.83 (0.55–1.26)	0.72 (0.46–1.11)	0.137
Model 2	1.00	0.79 (0.49–1.28)	0.70 (0.35–1.39)	0.296
Model 3	1.00	0.79 (0.49–1.27)	0.67 (0.33–1.35)	0.254
Plant Protein	*n* = 446(CKD = 73)	*n* = 451(CKD = 54)	*n* = 437(CKD = 36)	
Model 1	1.00	0.76 (0.51–1.14)	0.48 (0.30–0.75)	0.002
Model 2	1.00	0.63 (0.40–0.99)	0.34 (0.18–0.65)	0.001
Model 3	1.00	0.57 (0.35–0.91)	0.26 (0.12–0.56)	0.001
Animal protein	*n* = 439(CKD = 53)	*n* = 439(CKD = 57)	*n* = 456(CKD = 53)	
Model 1	1.00	1.00 (0.65–1.52)	0.89 (0.58–1.38)	0.630
Model 2	1.00	1.02 (0.66–1.59)	1.04 (0.62–1.75)	0.873
Model 3	1.00	1.01 (0.65–1.58)	0.98 (0.57–1.67)	0.965

*P* for trend across tertiles calculated with the exposure modelled as a continuous variable

Model 1: crude

Model 2: adjusted for age, sex, smoking, total energy intake, physical activity, BMI

Model 3: additionally adjusted for total fiber intake and energy percent from fat

## Discussion

In the present study, after adjusting for potential confounding variables, there was no significant association of total protein and animal protein intake with the incidence of CKD. However, we observed that participants in the highest tertile of plant protein intake decreased 70% risk of incident CKD compared to those in the lowest tertile. Even after excluding cases with diabetes or hypertension, the inverse associations between plant protein intake and incidence of CKD were seen, which shows the independency of our result to the well-known CKD risk factors. Considering the absolute amounts of plant protein to total protein, in higher tertiles of total protein intake, absolute amount of plant protein was higher, while the percentage of plant protein to total protein was lower. Since plant protein is not independent of total protein “the percentage of plant protein” in total protein intake is a more important variable than the absolute amount.

Because a high intake of animal protein increases renal damage in experimental animal models [24], the effect of low-protein diets on eGFR decline in humans has been of interest. To date, a few studies have investigated the association of long-term dietary protein sources with the risk of CKD. Lin et al. showed that higher intakes of animal fat and two or more servings of red meat per week were associated with kidney dysfunction [25]. In accordance to our finding, a cross-sectional study by Yuzbashian et al. revealed that a higher intake of plant protein was significantly associated with a lower risk of prevalent CKD. However, more animal protein intake was significantly associated with a higher risk of developing CKD. They claimed that a 20-g increase in plant protein intake reduced the risk of developing CKD by 16% [7]. In the current study, we did not find associations between either total or animal protein intake and incidence of CKD after adjusting for covariates. Compared with the previous investigations, our study has the advantage of a 6.1-year follow-up for eGFR changes.

Animal protein consumption is mostly associated with a high intake of saturated fatty acids and sodium from red meat and processed meats, which is linked with an unhealthy lifestyle [7]. In our most recent study conducted on 4881 participants of the TLGS who were free of CKD, participants in the highest quartile of total red meat intake had 73% increased risk of incident CKD compared with those in the lowest quartile. Furthermore, the substitution of total red with plant-based sources of protein decreased risk of CKD [12]. Haring et al. examined the relationship between dietary protein sources and risk for incident CKD in a prospective cohort study of 11,952 adults followed up for 23 years. They found that red and processed meat was adversely associated with CKD risk, while nuts and legumes were protective against the development of CKD [13].

Several potential mechanisms can explain the association of dietary plant protein with kidney function. Higher plant protein and lower animal protein intake lead to consumption of higher proportions of glutamic acid, proline, phenylalanine, cysteine and serine. This difference in amino acids could be the reason for the different effects of plant and animal protein on kidney function [26]. It is reported that higher intakes of plant protein from gluten can reduce serum triacylglycerol, oxidized low-density lipoprotein (LDL) cholesterol and uric acid [27]. In a similar study, significant decrease in LDL cholesterol, total serum cholesterol and triglycerides was reported in higher intakes of soy protein [28]. Plant protein may be helpful to lessen oxidized-lipoprotein–induced glomerular damage and progression of CKD by reducing these serum lipids levels [29]. Plant-based sources of proteins are also rich in calcium, magnesium, potassium, and vitamin C, which were associated with lower dietary acid load and improvement in kidney function [30]. These explanations can support the findings of the current study.

One of the limitations of this study was whether the results are related to plant protein itself or to other factors associated with more plant-based diets that are hard to establish. On the other hand, because CKD is a multifactorial illness, many genetic and environmental factors are involved. In the present study, while considering some confounding factors, it was not possible to modify them all. As in most epidemiologic studies, another limitation is our definition of CKD, which was established from limited number of isolated creatinine measurements. To confirm a chronic decrease in GFR, repeated measurements within 3 months are needed. Third, data on the proteinuria, uric acid, cystatin C, albumin, nephrotoxic medications and hospitalization of the participants were not considered. Fourth, the method which was used to measure creatinine was not calibrated by isotope dilution-mass spectrometry (IDMS) method. Another limitation of this study is that there is no data regarding the race of participants; thus, we could not be able to specify their proportions in our study.

## Conclusion

In summary, the consumption of more plant protein instead of animal protein seems to have possible preventive effects on CKD incident. Furthermore, considering the lack of association between dietary total protein intake and incident CKD may highlight the importance of sources of protein rather than the quantity of consumed protein.

## Data Availability

The data set is the property of Research Institute for Endocrine Sciences (RIES) and is made available upon approval of the research proposal by the research council and the ethics committee.

## Abbreviations

BMI: Body Mass Index
BP: Blood Pressure
CIs: Confidence Intervals
CKD: Chronic Kidney Disease
CVs: Coefficients of Variation
FCT: Food Composition Table
FFQ: Food Frequency Questionaire
FPG: Fasting Plasma Glucose
GFR: Glumerolar Filtration Rate
IDMS: Isotope Dilution-Mass Spectrometry
LDL: Low-Density Lipoprotein
MAQ: Modifiable Activity Questionnaire
MDRD: Modification of Diet in Renal Disease
MET: Metabolic Equivalent Task
OR: Odds Ratio
SPSS: Statistical Package for the Social Sciences program
TGs: Triglycerides
TLGS: Tehran Lipid and Glucose Study
USDA FCT: The United States Department of Agriculture Food Composition Database
